# Mechanism of Allosteric Modulation of the Cys-loop Receptors

**DOI:** 10.3390/ph3082592

**Published:** 2010-08-12

**Authors:** Yongchang Chang, Yao Huang, Paul Whiteaker

**Affiliations:** 1 Division of Neurobiology, Barrow Neurological Institute, St. Joseph’s Hospital and Medical Center, Phoenix, AZ 85013, USA; E-Mail: paul.whiteaker@chw.edu (P.W.); 2 Department of Obstetrics and Gynecology, St. Joseph’s Hospital and Medical Center, Phoenix, AZ 85013, USA; E-Mail: yao.huang@chw.edu (Y.H.)

**Keywords:** cys-loop receptors, ligand-gated ion channels, allosteric modulator

## Abstract

The cys-loop receptor family is a major family of neurotransmitter-operated ion channels. They play important roles in fast synaptic transmission, controlling neuronal excitability, and brain function. These receptors are allosteric proteins, in that binding of a neurotransmitter to its binding site remotely controls the channel function. The cys-loop receptors also are subject to allosteric modulation by many pharmaceutical agents and endogenous modulators. By binding to a site of the receptor distinct from the neurotransmitter binding site, allosteric modulators alter the response of the receptors to their agonists. The mechanism of allosteric modulation is traditionally believed to be that allosteric modulators directly change the binding affinity of receptors for their agonists. More recent studies support the notion that these allosteric modulators are very weak agonists or antagonists by themselves. They directly alter channel gating, and thus change the distribution of the receptor across multiple different affinity states, indirectly influencing receptors’ sensitivity to agonists. There are two major locations of allosteric modulator binding sites. One is in subunit interfaces of the amino-terminal domain. The other is in the transmembrane domain close to the channel gating machinery. In this review, we also give some examples of well characterized allosteric binding pockets.

## 1. Introduction

**Figure 1:**
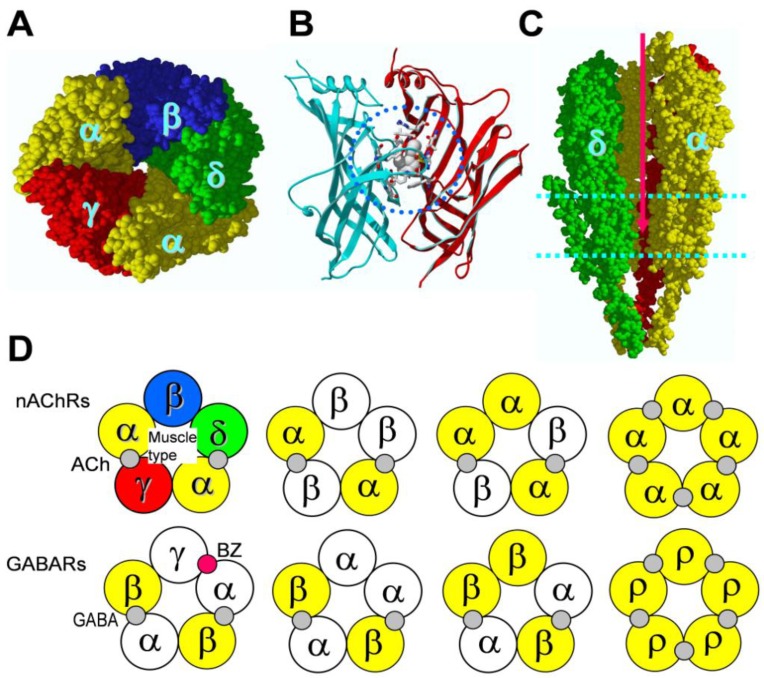
Structures of prevalent cys-loop receptors and locations of agonist binding sites. (A) Top view of the EM structure of the *Torpedo* nicotinic receptor, generated from 2BG9 PDB file [16], showing pentameric structure; (B) Side view of crystal structure of AChBP with nicotine bound in the subunit interface, generated from 1UW6 PDB file [17]. The nicotine is surrounded by residues that form the agonist binding pocket (within blue dashed circle); (C) Side view of the EM structure of the *Torpedo* nicotinic receptor with β subunit removed, revealing central vestibule and channel pore (red arrow), generated from 2BG9 PDB file [16]; (D) Location of agonist binding sites in different subunit interfaces in major subtypes of nicotinic and GABA_A/C_ receptors. Note that most heteromeric receptors have two orthosteric agonist binding pockets. In some cases (heteromeric α1β glycine receptor [29], α4β2 nicotinic receptor [30]), functional studies suggest a possible third neurotransmitter binding site. In addition, the αβγ GABA_A_ receptor has one allosteric benzodiazepine binding site (BZ).

The cys-loop receptor family is a major family of ligand-gated ion channels (LGICs). All members of this receptor family have a signature cysteine loop, two cysteine residues separated by 13 other residues, in the amino-terminal domain of each subunit. This family includes subfamilies of nicotinic receptors (nAChRs) [1], serotonin receptor type 3 (5-HT_3_R) [2], γ-aminobutyric acid receptors type A and C [3,4,5] (GABA_A/C_R), glycine receptors [6], zinc-activated cation channel [7], and invertebrate glutamate/serotonin-activated anionic channels or GABA-gated cation channels [8,9,10]. Recently, prokaryotic proton-gated ion channels have also been considered to be in the same family, although they do not have signature cysteines in the corresponding loop region [11,12,13]. In eukaryotic ligand-gated ion-channels, each receptor subunit has a large amino-terminal extracellular domain, four transmembrane domains (M1–M4) with the second transmembrane domain (M2) lining the pore, and a large intracellular loop between M3 and M4 [14,15]. Prokaryotic channels have similar subunit topology, but with a short M3–M4 intracellular loop. The receptors in this family have a pentameric structure. That is, five subunits form a pseudo-symmetrical receptor with a central vestibule (Figure 1A). Different combinations of subunits, and their isoforms, create structural diversity and thus different subtypes within each cys-loop receptor subfamily. Since each subtype has specialized function, the structural diversity also provides opportunities for subtype-selective drug development. The neurotransmitter binding sites (orthosteric sites) are located in the amino-terminal domain at subunit interfaces [15,16]. Figure 1B shows the crystal structure of nicotine in the binding site of the homologous protein, acetylcholine binding protein (AChBP) [17]. AChBP is homologous to the amino-terminal domain of cys-loop receptor’s subunits [18]. It also has a pentameric structure [18] and can bind nicotinic receptor agonists and antagonists [17,19,20]. Thus, AChBP crystal structures provide the best structural models for the amino-terminal domain and orthosteric binding sites. In the center of the transmembrane region of all cys-loop receptors is the ion conducting pore (Figure 1C) [15], which is controlled by neurotransmitter binding to orthosteric sites in the distal amino-terminal domain. Thus, the cys-loop receptors are allosteric proteins; binding of a neurotransmitter to the extracellular amino-terminal domain of its receptor allosterically controls the distantly located transmembrane ion channel pore, a process termed allosteric activation [21,22]. This allosteric activation occurs through an evolutionarily conserved allosteric network that connects orthosteric binding sites to the channel gate [23]. The detailed activation mechanism of cys-loop receptors has been extensively reviewed recently [21,24,25,26,27,28]. Figure 1D shows the neurotransmitter binding sites located at subunit interfaces of major subtypes of nicotinic receptors and GABA_A/C_ receptors. A typical heteromeric receptor (containing at least two different subunits) has two orthosteric binding sites. The remaining subunit interfaces of the amino-terminal domain of heteromeric receptors can potentially act as allosteric modulator binding sites. In fact, the well-characterized benzodiazepine binding site in a typical αβγ GABA_A_ receptor is located in the amino-terminal domain αγ subunit interface, homologous to the orthosteric GABA binding sites at the βα interfaces (Figure 1D). A typical homomeric receptor (with identical subunits), such as α7 or α9 nAChR, ρ1 GABA receptor, 5-HT_3_A receptor, or glycine α1 receptor, has five potential orthosteric binding sites. 

The function of the cys-loop receptors can be modified by allosteric modulators. By binding to the receptor at an allosteric site, a site distinct from the orthosteric neurotransmitter binding sites, allosteric modulators alter the agonist sensitivity (shift of concentration-response curve), agonist efficacy (maximum response), and channel gating kinetics for activation and desensitization. Note the difference between allosteric activation and allosteric modulation. For allosteric activation, the binding of neurotransmitter to the “orthosteric” binding site (allosterically) controls the distantly located gating machinery. However, for allosteric modulation, modulator binding to additional “allosteric” sites modifies the action of agonists binding to the orthosteric sites. That is, the modulator allosterically alters the orthosteric agonist’s action We will discuss kinetic models and mechanisms of allosteric modulation in section 3, and well-characterized allosteric sites in section 3. 

## 2. Kinetic Models of Allosteric Activation

**Figure 2:**
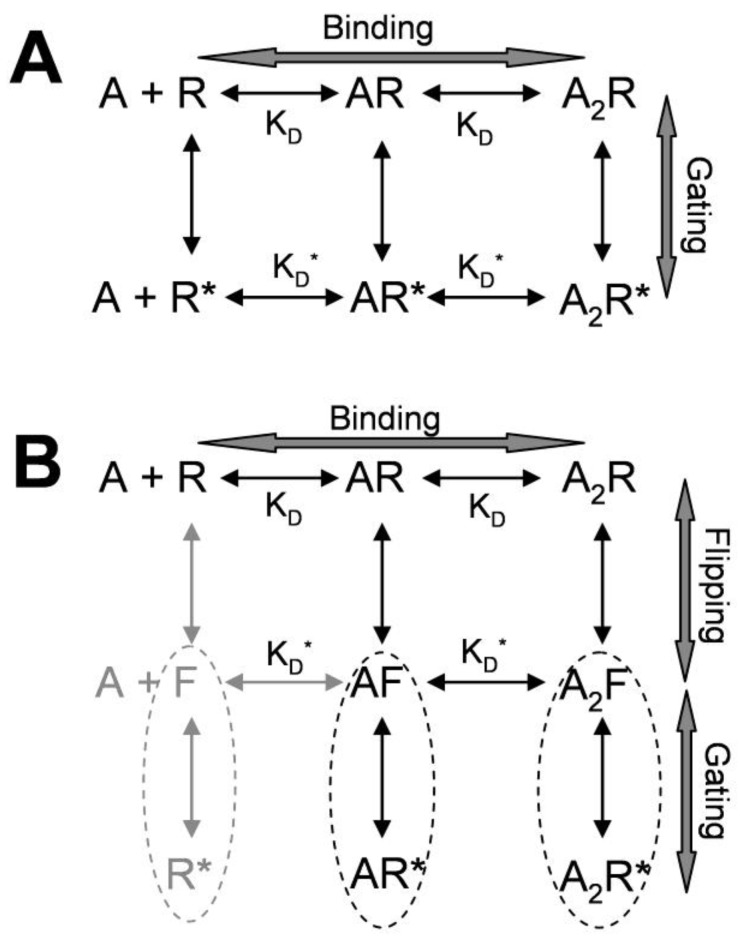
Kinetic schemes for allosteric activation of a typical heteromeric cys-loop receptor. (A) Monod-Wyman-Changeux allosteric activation (MWC) model for allosteric activation [22,33], R, AR, A_2_R are receptors in the resting states with 0, 1 or 2 agonist bound. They have low binding affinity (K_D_). R*, AR*, A_2_R* are receptors in open states with high binding affinity (K_D_*) for agonist; (B) Refined MWC model with intermediate high affinity flip states F, AF, and A_2_F [34]. Flip states are pre-open states with increased affinity for agonists, suggesting a conformational changes in the binding site. Thus, flip and open states in each oval may be considered “activated” states.

The activation mechanism of the nicotinic receptors can be understood in terms of the Monod-Wyman-Changeux allosteric activation (MWC) model [8,22,31]. This model can explain the spontaneous openings observed in the wild type nicotinic receptor [32]. Similarly, activation of the wild type, and spontaneously-opening gating mutant, GABA_A_ receptors can be well fitted by the MWC model (Figure 2A). 

An important feature of this model is that the receptor has low affinity (K_D_) resting closed states and high affinity (K_D_*) open states [22,33]. Binding of the receptor to its agonist facilitates transitions from low affinity states to high affinity states. This model can explain how mutations in the channel gate make the receptor more sensitive to agonist. That is, loosening the channel gate would increase spontaneous openings, resulting in more receptors in the high affinity states, and thus increasing sensitivity of the receptor to agonists. This mutational effect is similar to the effects of positive allosteric modulators (see below). The MWC model was further refined by the finding that partial and full agonist-induced openings have similar opening and closing rates, but with a major difference in equilibrium distribution between resting state and “flipped” state, a pre-opening intermediate state with higher affinity. For a full agonist, the equilibrium constant for flipping is large, so that most fully liganded receptors will shift to the flip state. For a partial agonist, the equilibrium constant is small, so that a smaller fraction of fully-liganded receptors will transit to the flip state. However, once the receptor transitions to a flipped state, full and partial agonists will gate the channel similarly. Thus, there is a high affinity “flip” state between low affinity resting state and high affinity open state (Figure 2B;[34]). The pre-opening flip state is an agonist-induced conformational state preceding channel opening. In this sense, the flip state and open state of the receptor could be collectively considered as “activated states” (as enclosed in the dashed ovals in Figure 2B). Note that in the kinetic scheme of Figure 2B, there is no connection between open states. This is because association to, and dissociation from, the open states are rare events, and adding connections between open states does not produce any detectable improvement in fitting [29]. Thus, agonist association and dissociation rates for the open states are much smaller than those for binding in the flip state or resting state. The molecular basis for the scarcity of transitions between open states is still not clear. One possible explanation is that the open states could have even higher affinity than the flip states, so that further binding pocket closure would limit association and dissociation. Detailed descriptions of the evolving kinetic and activation mechanism models can be found in several recent review papers [21,24,25,26,27].

In summary, kinetic studies revealed that binding and gating are coupled, with mutual influence. The receptor’s affinity for agonists is state dependent. Alteration of the receptor distribution across different conformational states, either by mutation or by allosteric modulator binding, alters the distribution of receptors across different agonist affinity states. These alterations change the apparent affinity of the receptor for its agonists. This concept forms the foundation of allosteric modulation.

## 3. Kinetic Models and Mechanisms of Allosteric Modulation

**Figure 3:**
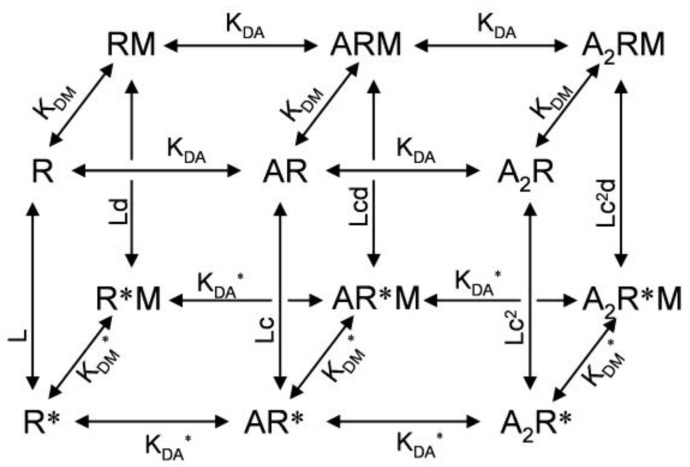
Kinetic scheme for allosteric activation and modulation mechanisms (adapted from [35]). Front is the conventional MWC model. The only difference is that K_DA_ and K_DA_* are used to represent agonist binding affinity. Allosteric modulator (M) bound states are shown at the back of the figure, with K_DM_ and K_DM_* describing two different affinities for the modulator in resting or activated states. L is the ratio between R and R*. c is the ratio between K_DA_* and K_DA_. d is the ratio between K_DM_ and K_DM_*.

For the mechanism of allosteric modulator action, it has long been believed that binding of allosteric modulators to the receptor directly changes receptor affinity for its agonist. In this model, the allosteric modulator does not have an intrinsic ability to open the channel. However, this view has been changed by the observation that the benzodiazepine allosteric agonist, diazepam, can directly activate the wild type GABA_A_ receptor (with very low efficacy). This effect is only visible when the receptor is highly expressed in *Xenopus* oocytes [35]. The super-high expression of the wild type GABA_A_ receptor also makes it possible to observe spontaneous opening, allowing for testing of the direct action of GABA_A_ receptor negative allosteric modulators. In fact, the benzodiazepine inverse agonist methyl-6,7-dimethoxy-4-ethyl-β-carboline-3-carboxylate (DMCM) can inhibit the measurable current formed by spontaneous opening of highly expressed wild type GABA_A_ receptors. These small fractional changes in channel opening induced by positive or negative allosteric modulators can account for the alteration of the receptor sensitivity to agonist according to the MWC allosteric activation mechanism [35]. A similar direct gating mechanism, termed allosteric coagonism, was also proposed for the mechanism of benzodiazepine action. This view is supported by the observation of direct activation and inhibition of a mutant GABA_A_ receptor by benzodiazepine agonists and inverse agonists [36]. By making concatenated subunits and selectively destroying one orthosteric binding site, Baur and Sigel found that the benzodiazepine allosteric potentiator diazepam can facilitate channel opening when the receptor has only one orthosteric binding site [37]. This finding also supports the view that allosteric modulators facilitate channel gating, and that apparent agonist affinity changes are due to this gating effect. Normally, in the resting unbound state, GABA_A_ receptors have very low open probabilities (10^−4^–10^−5^) [33,36]. The receptor with a single agonist bound will have an open probability increase of ~700 fold [33]. However, the open probability of a singly bound receptor is still very low. Binding of a positive allosteric modulator to the receptor will further increase open probability as observed by Baur and Sigel. The kinetic model in Figure 3 represents an expanded MWC model that includes direct gating effects of GABA_A_ receptors by a modulator [35]. In this model, direct gating by a positive allosteric modulator also increases the agonist binding affinity. In fact, the benzodiazepine agonist flurazepam can induce structural changes in the orthosteric GABA binding pocket, further supporting a direct gating mechanism for allosteric modulators [38].

This co-activation mechanism of GABA_A_Rs by benzodiazepines seems to be applicable to other allosteric modulators acting on different sites. For example, the general anesthetic etomidate potentiates GABA_A_ receptor agonist responses at low concentrations, but directly activates the GABA_A_ receptor at high concentrations. At the same time, high concentration etomidate also further shifts the GABA concentration-response curve to the left. Both potentiation and activation effects can be well described by a single MWC kinetic model [39], similar to that for benzodiazepines. Affinity labeling has identified the binding site of etomidate in the transmembrane domain (Met236 in the M1 domain of α1 subunit and Met286 in M3 domain of β2 subunit) [40]. The allosteric effect of etomidate can be mimicked by two substitutions at these sites with the bulky residue tryptophan [41]. In contrast, the benzodiazepine binding pocket is located in the amino-terminal domain (see section 4.1). Thus, regardless the location of the binding site, in the amino-terminal domain distant from the channel gate or in the transmembrane domain close to the channel gate, the binding of allosteric modulators to their sites alters the channel gating. Positive allosteric modulators reduce the gating energy barrier to facilitate channel opening, serving as weak co-agonists (binding to a site that is distinct from the orthosteric neurotransmitter binding site). Conversely, negative allosteric modulators increase the gating energy barrier to inhibit channel opening. For example, ρ1 GABA_C_ receptor function can be inhibited by several neurosteroids [42]. These neurosteroids inhibit the GABA_C_ receptor allosterically as suggested by monitoring conformational changes with site-specific fluorescence techniques [43]. However, the mechanisms of GABA_C_ receptor inhibition by individual neurosteroids are not the same. Functional inhibition by β-estradiol is correlated to the inhibition of the GABA-induced fluorescence changes in multiple locations of the amino-terminal domain, including at K217C in loop F, L166C in loop E, and L66C away from the binding loops, [43]. This suggests that β-estradiol can antagonize GABA-induced conformational changes. In contrast, pregnanolone, pregnanolone sulfate, allo-pregnanolone sulfate, and tetrahydrodeoxycorticosterone influence GABA-induced fluorescence changes differently, suggesting distinct detailed structural mechanisms [43]. Note that some competitive antagonists could be very weak agonists or inverse agonists. However, since they compete with agonist for the same binding site, their modulatory effect on the agonist response could only be seen at very low concentrations when the agonist concentration is also very low, with low probability of mutual competition. In any case, since these competitive antagonists compete with agonists for the same orthosteric binding site, they are not allosteric modulators. 

Since nicotinic receptors have an allosteric activation mechanism similar to that for GABA_A_ receptor [22], they likely have a similar allosteric modulation mechanism. In fact, the allosteric modulator PNU-120596 for α7 nicotinic receptor has its binding site in the transmembrane domain [44] (see Section 4.2 for details), but it can induce a similar, though not identical, conformational change along the activation pathway as acetylcholine-induced conformational changes during channel activation [45]. Similarly, allosteric modulation by divalent cations of nicotinic receptors involves conformational changes in the amino-terminal domain, partially shared with agonist-induced conformational changes [46]. These experimental observations further support a co-agonist action for positive allosteric modulators in nicotinic receptors.

In glycine and GABA receptors, alcohol size cut-off (see Section 4.2 for a fuller explanation) is related to the size of the sidechain of two residues in the transmembrane domains (M2 and M3, see Section 4.2 for details), suggesting a transmembrane location of their binding site. However, the structure of loop 2 from the amino-terminal domain, which plays an important role in coupling agonist binding and channel gating, is also critical for ethanol’s effect [47,48]. Whether loop 2 and its surrounding residues form a second binding site for ethanol is still not clear. Ethanol’s modulatory effect on glycine receptor can also be influenced by a third site within the intracellular loop between M3 and M4, which modifies ethanol’s effect by interacting with G protein subunits [49]. Thus, it is likely that the allosteric modulation of receptor function by ethanol is through co-agonism with ethanol binding to one site in the M2 and M3 domains, and that this co-agonist effect can itself be allosterically modulated by other allosteric modulators, such as G protein interacting with the M3–M4 site, or by mutations of important residues along the activation pathway, such as in loop 2. 

In summary, several lines of evidence suggest that allosteric modulation of cys-loop receptor function is mediated mainly through a weak direct gating effect by allosteric modulators. 

## 4. Binding Sites for Allosteric Modulators

As just described, allosteric modulators of cys-loop receptors can bind in two major locations. The first location is at subunit interfaces of the amino-terminal domain, homologous to the orthosteric agonist binding sites, but at a different subunit interface. Benzodiazepine agonists, partial agonists, antagonists, partial inverse agonists, and inverse agonists bind to sites in this category. We will describe the amino-terminal binding sites for allosteric modulators in Section 4.1. The second major location of allosteric modulator binding sites is in the transmembrane domain near the channel gate. Barbiturates, neurosteroids, alcohols and general anesthetics bind to sites of this type. We will describe the transmembrane binding sites with several examples in Section 4.2. Noncompetitive antagonists also bind to the transmembrane domain. Some of them have binding sites within the pore. Thus, binding of these antagonists will occlude the channel pore. The effects of these channel blockers are usually use-dependent. That is, the binding site is only accessible in the open state. Other noncompetitive antagonists allosterically inhibit channel activity. It is also possible that a single antagonist can block the pore, and simultaneously allosterically inhibit channel function. This may explain a small shift in concentration-response curve sometimes observed in the presence of noncompetitive antagonist. Picrotoxin is an example of a pore blocker, although the mechanism of its non-competitive antagonism is still not fully understood. Three studies suggest that it can actively antagonize conformational changes induced by GABA in the amino-terminal domain; suggesting that, in addition to blocking the pore, picrotoxin also negatively modulates channel gating and facilitates channel closing [43,50,51]. 

In addition to these two major binding sites, residues in the coupling region between the agonist binding site and the channel domains may also be involved in mediating allosteric modulation. For example, mutations of the Gly219 residue in the pre-M1 region of the GABA_A_ receptor β2 subunit can abolish the effect of several allosteric modulators, such as barbiturates, alphaxalone, etomidate and propofol [52]. The pre-M1 residue Arg246 of the 5-HT3A receptor is also an important determinant for allosteric modulation [53]. Another example is that the zinc modulation of GABA_A_ receptors involves two charged residues located in the cys-loop region [54] that plays an important role in coupling the amino-terminal domain to the channel gate [55,56]. 

### 4.1. Amino terminal binding sites for allosteric modulators

**Figure 4:**
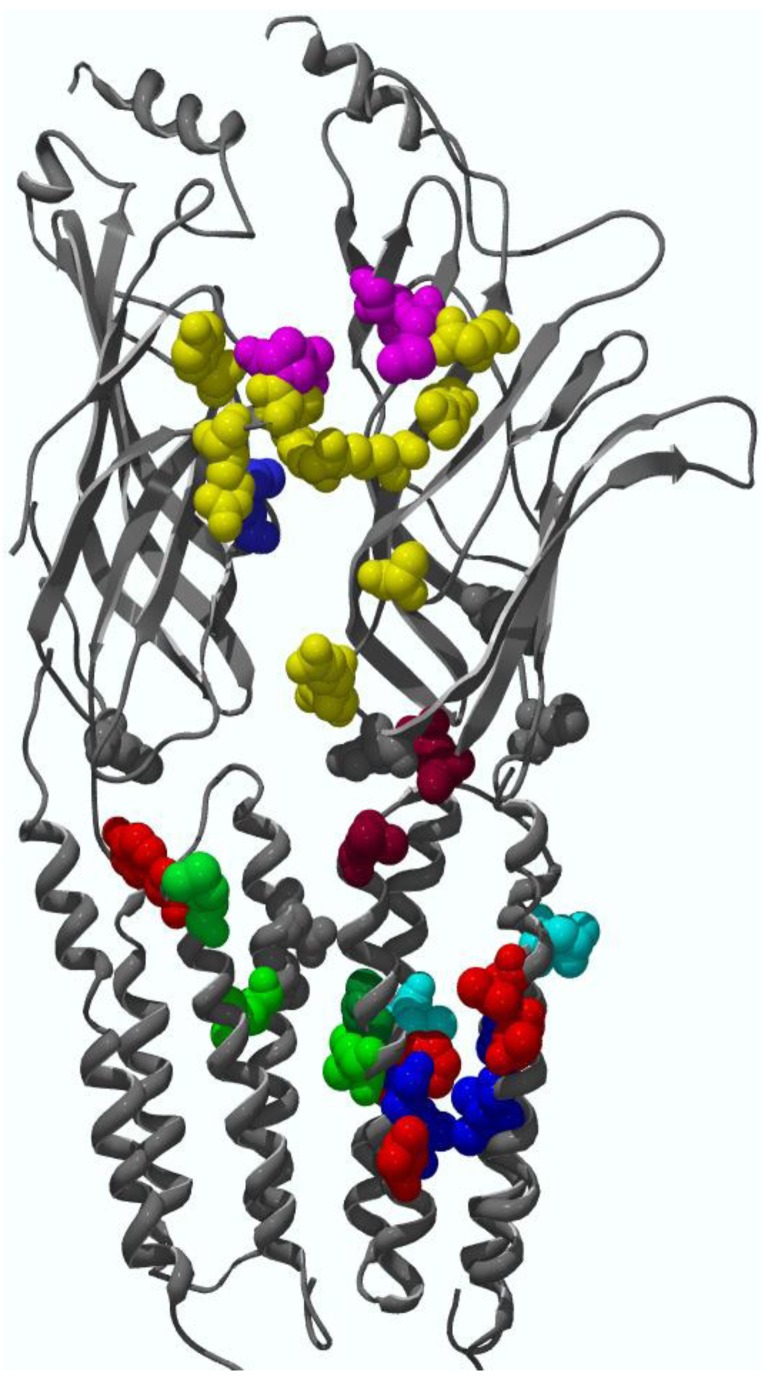
Allosteric modulator binding sites (shown as residues with side-chains displayed) mapped on to the homologous sites of the EM structure of *Torpedo* nicotinic receptor α (right) and β (left) subunits (made from 2BG9 PDB file [16]). A wide range of binding sites for different allosteric modulators are color-coded: Yellow: benzodiazepine sites; Pink: morantel sites; Red: endogenous neurosteroid sites; Green: etomidate sites; Dark green: volatile anesthetic site; Blue: PNU-120596 site for α7 nicotinic receptor; Cyan: overlapped sites in M2 or M3 domains shared by multiple modulators; Grey: divalent cation sites; Burgundy: pre-M1 residues that influence anesthetic modulation in GABA_A_ receptor or 5-HT3 receptor; Note that if a binding site residue is located within a subunit, then the residue is mapped to the α subunit (right). If a binding pocket is in subunit interface (involving both subunits), then the residues are mapped to the positions in α or β subunits, so that they are facing to the same subunit interface. The G protein binding site in the GlyR intracellular loop is not shown, since much of the intracellular loop structure is undefined in the EM structure used to create this illustration.

Since most heteromeric cys-loop receptors have only two orthosteric binding sites for agonists, there is a lot of potential for future drug development in the remaining subunit interfaces that do not bind conventional agonists. The best-characterized example of an allosteric site in amino-terminal domain is the benzodiazepine binding pocket of GABA_A_ receptors. It is located in the α−γ subunit interface, homologous to the GABA binding site in the β−α subunit interface (Figure 1D). The benzodiazepine binding pocket is formed by residues in six binding loops, designated as loops A through F, and homologous to those that form the orthosteric binding pockets. Residues (with rat α1 subunit numbering) identified from Loops A (His101 [57,58], B (Tyr159 [59]), and C (Gly200 [60], Thr206, and Tyr209 [59,61]) are contributed by the α subunit and form the principal face of the binding pocket. Residues in loops D (Phe77 [62,63], Ala79, Thr81 [64,65]), E (Met130 [63,66]), and F (Glu189, Thr193, and Arg194 [67]) are contributed by γ2 subunit and form the complementary face of the binding pocket (Figure 4). 

For nicotinic receptors, a recent study shows that there is an amino-terminal noncanonical allosteric site for the positive allosteric modulator morantel in the α3β2 subtype [68]. This binding site for the allosteric modulator is located in the β−α subunit interface (Figure 4), in contrast to the α−β interface for the ACh binding site (Figure 1D). Note that binding residues identified for morantel are located what is equivalent to the upper half of the homologous orthosteric binding pocket. 

### 4.2. Transmembrane domain binding sites for allosteric modulators

The transmembrane domain is the channel forming domain, which includes gating machinery to control channel opening and closing. Several classes of allosteric modulators have their binding pockets in the transmembrane domain. These include ethanol and other short-chain alcohols, neurosteroids, barbiturates, general anesthetics, and some α7 nAChR positive allosteric modulators (described more-fully later). A general feature of this type of modulation is that the modulators bind to a cavity near the channel pore-lining domain, M2, and more directly influence channel gating. The following are some examples of allosteric modulator binding sites in this category. 

Binding of alcohol and volatile anesthetics to the receptor has a cutoff phenomenon. That is, the allosteric effect is dependent on the size of these compounds. When the size of the compounds increases above a certain level, the allosteric effect either decreases or is abolished. This cutoff phenomenon suggests that the receptor has a cavity with a defined volume to accommodate this type of allosteric modulator. For example, using three volatile anesthetics combined with site-directed mutagenesis at the 232, 270 and 279 positions of human GABA_A_ receptor α2 subunit to residues with different sizes, Jenkins *et al*. estimated the size of the binding cavity for these anesthetics as between 250 and 370 Å^3^ in the wild type GABA_A_ receptor [69]. Thus, the residues Leu232 (M1), Ser270 (M2, 15’ position), and Ala291 (M3, homologous to β2Met286) in the α2 subunit play an important role in defining the binding cavity for these small molecules. For the alcohol cutoff, the effect of an alcohol is dependent on the length of its carbon chain. When the carbon chain of an alcohol exceeds a given length, its effect will decrease. The cutoff value for the carbon chain length of alcohols varies with different receptors. For example, the homomeric α1 glycine receptor has an alcohol cutoff value of 10, whereas the homomeric ρ1 GABA receptor has a cutoff value of 7. Using chimeras of glycine and GABA receptors, Wick *et al*. identified two critical residues in M2 and M3 domains as the main determinants of the observed difference in cutoff [70]. These two residues are Ser267 (M2, 15’ position) and Ala288 (M3) in the glycine receptor α1 subunit. The homologous residues in the ρ1 GABA receptor are Ile307 (M2) and Trp328 (M3, homologous to β2Met286). Mutation at Ser267 of the glycine receptor α1 subunit to a larger sized residue decreases the cutoff, whereas mutations of Ile307 and Trp328 in the ρ1 GABA receptor subunit to smaller residues increases the cutoff, supporting the concept of a defined cavity between M2 and M3 domains for alcohol binding. 

Non-volatile general anesthetics, such as etomidate, propofol and barbiturates are mainly positive allosteric modulators of GABA_A_ receptors, although they can inhibit nicotinic receptors and influence other channel function [71]. The binding sites of these allosteric modulators are also located in the transmembrane domain. In GABA_A_ receptors, the etomidate binding site was identified by photoaffinity labeling [α1Met236 (M1), β2Met286 (M3)] [40] and by a mutagenesis study [β2/β3Asn265 (M2, 15’ position)] [72]. The M3 binding site Met286 is shared with propofol [73], whereas M2 residue Asn265 is homologous to Leu270 of the 5-HT3 receptor, which is also responsible for volatile anesthetic action [74]. ([H^3^]azi)Etomidate photoaffinity labeling of α1Met236 (M1) &β2Met286 (M3) can be directly competed by volatile anesthetic isoflurane [75], suggesting they share the binding site. However, ([H^3^]azi) etomidate photoaffinity labeling is allosterically inhibited by barbiturates and propofol [75], and allosterically enhanced by neurosteroids [76], suggesting these allosteric modulators can directly induce conformational changes in the transmembrane domains of the receptor and thus alter the binding of other allosteric modulators. 

Neuroactive steroids, termed neurosteroids, include steroid hormones, their metabolites, and synthetic steroid anesthetics. In addition to genomic effects (regulating gene expression), many steroid hormones and their metabolites have non-genomic effects, including allosteric modulation of ligand-gated ion channel function. Naturally occurring neurosteroids are endogenous regulators of GABA_A_ receptors [77]. A good example of non-genomic neurosteroid effects is positive allosteric modulation of GABA_A_ receptors by the endogenous neurosteroids allopregnanolone and tetrahydrodeoxycorticosterone (THDOC). At low concentrations, they potentiate GABA effects, whereas at high concentrations, they directly activate GABA_A_ receptors. These two different effects are mediated by binding of the neurosteroids to two different binding sites in the transmembrane domain [78]. The potentiation site is located within the transmembrane domain of the α1 subunit, formed by residues Gln241 (M1), Asn407 (M4), and Tyr410 (M4). In contrast, the activation site is located at the subunit interface of the transmembrane domains between β2 and α1 subunits, formed by β2Tyr284 (M3) and α1Thr236 (M1). Neurosteroids also modulate nicotinic receptors. However, they are not well characterized in this context. 

Compared to GABA receptors, development of nicotinic receptor allosteric modulators is more recent. Positive allosteric modulators for the α7 nAChR are the best-developed examples of nicotinic receptor modulators. α7 nAChR has fast desensitization kinetics. Positive allosteric modulators (PAMs) for α7 nAChR can be classified into two types: type I and type II, according to their influence on current kinetics. Type I PAMs mainly potentiate current without significantly influencing receptor desensitization. Ivermectin [79], 5-hydroxyindole (5-HI) [80], genistein [81], compound 6 [82], and NS-1738 [83] are examples of type I PAMs. 5-hydroxyindole is also an allosteric modulator for serotonin receptor type 3. The binding site of 5-HI in 5-HT3A receptor is located at Leu293 of the M2 domain [84]. In contrast, type II PAMs can dramatically reduce desensitization or even re-activate desensitized receptors. PNU-120596 is the best-characterized representative of this type [85]. Its binding pocket is located in the transmembrane domain [44], and is formed by five residues: Ser222 (M1), Ala225 (M1), Met253 (M2, 15’ position), Phe455 (M4), and Cys459 (M4). Each α7 subunit harbors one such site.

Other allosteric modulators: In addition to small drug compounds, cys-loop receptors can be allosterically modulated by divalent cations, such as zinc and calcium, and some small peptides. For example, zinc can inhibit GABA_A_ [86] and GABA_C_ receptors [87]. Calcium can modulate nicotinic receptor desensitization kinetics [88]. Secreted mammalian Ly-6/uPAR-related protein 1 (SLURP-1) is also a potentiator of α7 nicotinic receptors [89]. Small peptide allosteric modulators of glycine receptors can be selected from a peptide phage-display library [90]. The zinc binding sites for zinc modulation of GABA_A_ receptors have been identified as five charged residues in three locations of the receptor. They are α1Glu137 (pre-cys-loop), α1His141 (cys-loop), β3Glu182 (loop F), β3His267 (M2, 17’ position), β3Glu270 (M2, 20’ position) [54]. Similar sites in the β2 subunit are responsible for the high sensitivity of the α1β2 GABA_A_ receptor to zinc potentiation [91]. The calcium binding sites in α7 nAChR are at Glu172 of loop F, homologous to the zinc binding site in the β3 GABA receptor subunit (β3Glu182), and Glu44 of loop 2 [92,93]. 

In summary, the mechanism of action for all allosteric modulators of cys-loop proteins is likely to involve altering channel gating. The alteration of channel gating will influence the distribution of receptors across different affinity states. The net observable effects are changes in the receptor maximal current, gating kinetics, and sensitivity to agonist. All these changes can be explained by a single MWC-type allosteric model. There are two major binding site locations for allosteric modulators: at amino-terminal domain subunit interfaces and the transmembrane domain. However, other locations along the activation pathway, especially the coupling region between amino-terminal domain and transmembrane domain, or even the intracellular loop, could be potential sites for novel allosteric modulator development. In fact, G protein βγ can modify glycine receptor function by interacting with two basic motifs in the beginning and the end of the M3−M4 intracellular loop [94]. The positively charged residues in these two motifs, along with the Gβγ protein, are also critical for the action of ethanol on the glycine receptor [49]. Figure 4 summarizes the binding sites discussed above, mapped onto the 3-D EM structure of *Torpedo* nicotinic receptor α and β subunits [16]. 

GABA_A_ receptor allosteric modulators are relatively well characterized and developed. A recent research trend is to develop novel allosteric modulators with subtype specificity in other members of the cys-loop family, especially nicotinic receptors. Another important development is negative allosteric modulators of GABA_A_ receptors. Development of GABA_A_ receptor negative allosteric modulators was hampered by their non-selectivity between different receptor subtypes, resulting in increased seizure susceptibility. However, recent development of more subtype selective negative allosteric modulators shows promising results. For example, α5 subunit containing GABA_A_ receptors are mainly distributed in the hippocampus, and α5 selective inverse agonists (negative allosteric modulators) can improve cognitive function [95,96]. We expect more subtype specific allosteric modulators for cys-loop receptors will be developed in the near future.
